# Ureterocele Diagnosed With Point-of-Care Ultrasound

**DOI:** 10.7759/cureus.35283

**Published:** 2023-02-21

**Authors:** Edgar J Miranda Gonzalez, Nicholas Bencomo, Alfredo Tirado, Veronica Tucci

**Affiliations:** 1 Department of Emergency Medicine, HCA Healthcare/USF (University of South Florida) Morsani College of Medicine GME: Oak Hill Hospital, Brooksville, USA

**Keywords:** emergency department, ultrasound, sepsis, ureterocele, pocus

## Abstract

Sepsis is a major cause of mortality as a life-threatening condition that arises when the body’s response to an infection injures its own tissue, and in the past decade, emphasis has been placed on early treatment to decrease mortality. In this case, we discuss the presentation of a young patient with sepsis due to acute complicated pyelonephritis with an obstructing ureterocele diagnosed by point-of-care ultrasound and explore the use of point-of-care ultrasound in sepsis.

## Introduction

Sepsis is a common diagnosis in the emergency department, and the mortality associated with these cases appears to be related to early antibiotic administration as well as early procedural intervention, if necessary [1,2]. In patients with sepsis due to pyelonephritis, imaging studies become important for detecting conditions that must be corrected in order to prevent worse outcomes [3]. Obstruction in the urinary tract can be due to calculi, tumors, strictures, or anatomical abnormalities, which invariably will lead to hydronephrosis; an infected obstructive kidney is considered an indication for urgent urologic evaluation because mortality rates associated with septic shock could approach 40% [4]. Studies have shown a sensitivity of point-of-care ultrasound (POCUS) of 72-83.3% [1]. Even though there are multiple protocols for POCUS in the ED, such as the rapid ultrasound in shock and hypotension (RUSH), there are no specific protocols regarding sepsis [1,5]. In this case, we discussed how the use of POCUS on a young patient with sepsis helped us identify an obstructive ureterocele as the culprit allowing us to get the patient evaluated by urology.

## Case presentation

A 22-year-old female presented to the emergency department (ED) with a chief complaint of dysuria. She endorsed that symptoms have been progressively worsening for the past three days and today, are associated with low back pain as well as chills. She had concerns that her intrauterine device (IUD) was dislodged but denied vaginal bleeding or discharge and a history of urinary tract infections or sexually transmitted infections.

On initial evaluation, the patient was tachycardic at 127 beats per minute with a blood pressure of 107/58 mmHg, a respiratory rate of 22, and a temperature of 37.9°C via an oral thermometer. Initial physical examination was remarkable for suprapubic tenderness to palpation and right-sided costovertebral angle tenderness to percussion. There was no abdominal rebound or guarding appreciated on physical examination.

Due to tachycardia, tachypnea, and a concern for an infectious source, the hospital sepsis protocol was activated. Blood cultures and lactic acid were obtained and then, the patient was given intravenous ceftriaxone with a 20 ml/kg NS bolus. Laboratory results were remarkable for a white blood cell count of 14.9x109/L with urinalysis suggestive of urinary tract infection urinary tract infections, as there were positive nitrates, leukocyte esterase, >100 white blood cell count /hpf, and many bacteria. The patient's lactic acid was elevated at 2.5 mmol/L.

**Figure 1:**
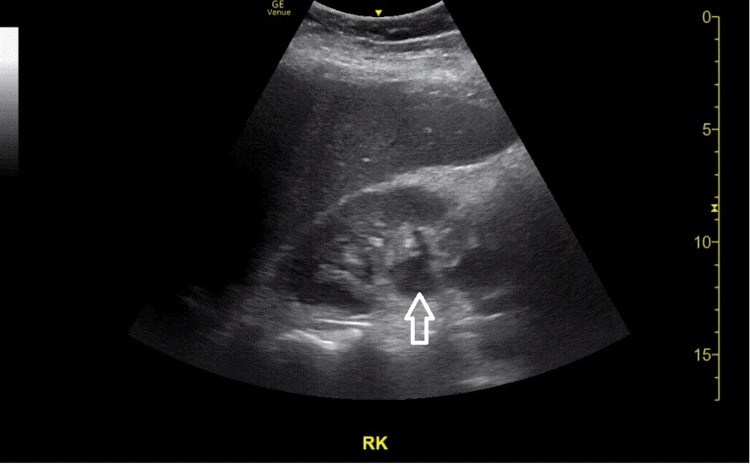
Longitudinal view of the right kidney showing an anechoic area in the renal pelvis (arrow) suggestive of hydronephrosis

**Figure 2:**
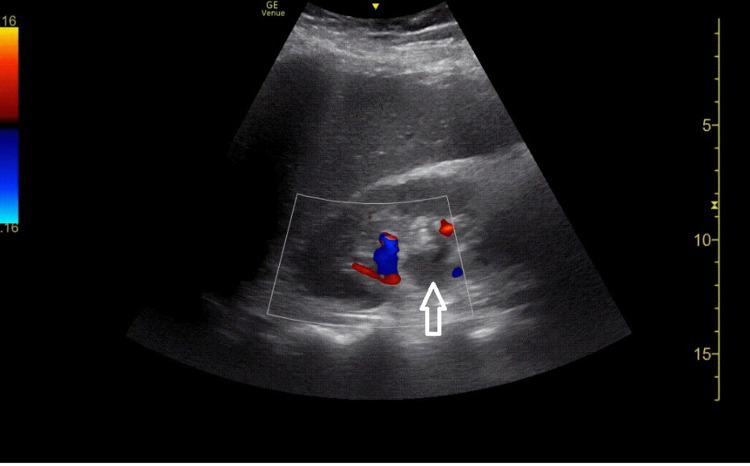
Longitudinal view of the right kidney with color flow showing the absence of flow on the anechoic renal pelvis (arrow) confirming mild to moderate hydronephrosis

As there remained a concern for sepsis with pyelonephritis as the likely source of infection, point-of-care ultrasound of the urinary system was performed. Views of the right kidney showed mild to moderate hydronephrosis (Figures 1, 2).

**Figure 3:**
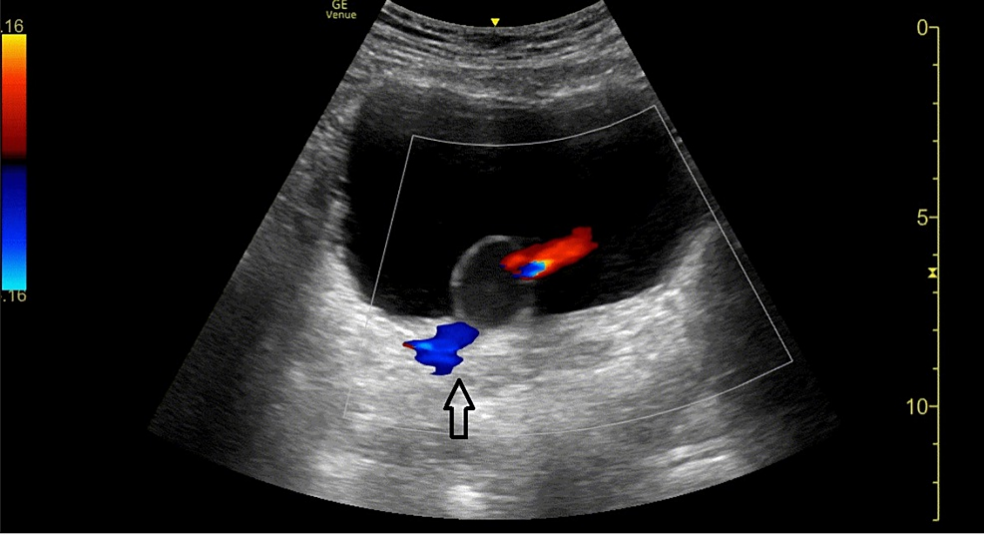
Transverse view of the pelvis showing an intravesical ureterocele arising from the right UVJ Color Doppler shows a partial flow of urine into the bladder but also regurgitation of urine at the right ureterovesical junction (arrow) UVJ: ureterovesical junction

**Figure 4:**
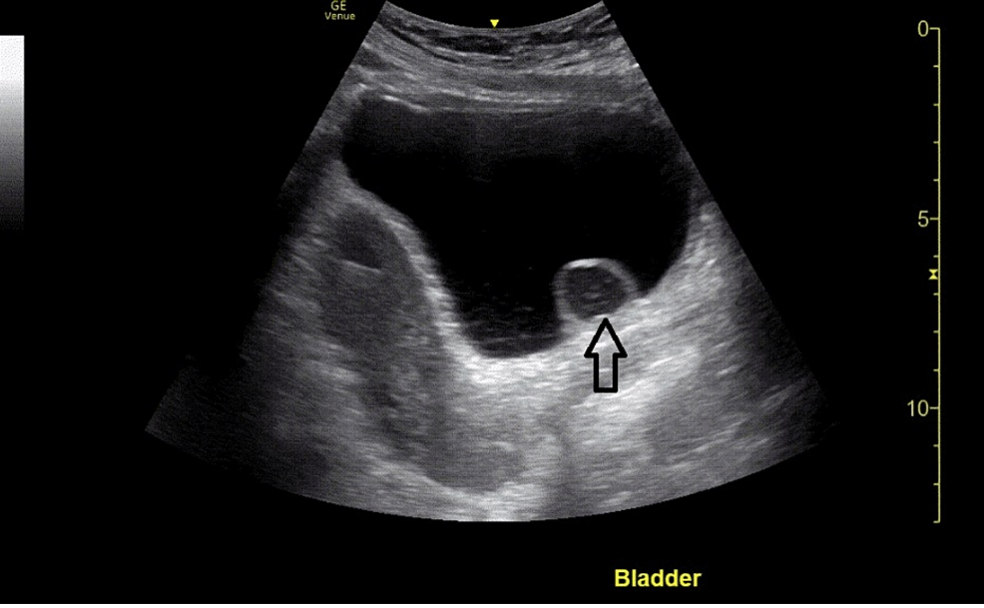
Longitudinal view of the pelvis showing the intravesical ureterocele in the posterior aspect of the bladder (arrow)

Bladder images displayed a cystic-like structure arising from the right ureterovesical junction expanding and contracting where color Doppler showed not only a partial flow of urine into the bladder but also regurgitation of urine at the right ureterovesical junction (Figures 3, 4). Computed tomography of the abdomen and pelvis located a 2.2 cm right ureterocele at the right ureterovesical junction causing hydroureteronephrosis. Again, the patient denied any history of any previous urinary tract infection diagnosis.

The patient was admitted to the medical floor with urologic consultation due to sepsis associated with the presence of a ureterocele in an adult patient. Urine cultures grew pan-sensitive *Escherichia* (*E.) coli*. A nuclear medicine renal flow scan demonstrated right-sided obstructive uropathy resulting in mild functional impairment.

Urology evaluated the patient and recommended oral antibiotics for 10 days followed by one month of low-dose regimen and outpatient follow-up for surgical consideration.

## Discussion

A ureterocele is an abnormal cystic dilation of the lower part of the ureter [6]. It is a condition most often associated with the pediatric population that is often diagnosed in the antenatal period by ultrasound, while in the postnatal period, it is usually diagnosed after evaluation of patients presenting with severe, febrile urinary tract infection in the first months after birth [6]. In adults, the diagnosis is usually made incidentally due to recurrent urinary tract infections and intermittent flank pain [7]. In our case, we can see the utility of point-of-care ultrasound to diagnose ureterocele in a young patient with urosepsis. This helped us to identify an anatomical defect in a patient, that if discharged, would have led to a high risk of adverse outcomes.

A 2016 study prospective study showed that point-of-care ultrasound was able to improve the sensitivity of a physician to identify the source of infection in septic patients by 25% [5]. Although there is an argument that point-of-care ultrasound will not assist in diagnosing sepsis, such as in cases of uncomplicated urinary tract infection, there seems to be a trend emerging that point-of-care ultrasound, as discussed above, can help detect cases of complicated urinary tract infection that may require surgical intervention. Additionally, there is a 2019 case report demonstrating the potential benefit of point-of-care ultrasound in undifferentiated sepsis in an elderly patient, who presented with nonspecific symptoms [1]. In this case, point-of-care ultrasound demonstrated obstructive pathology of the urinary system, prompting the recommendation for an algorithmic approach to sepsis utilizing point-of-care ultrasound early in a patient’s presentation [1].

Our case is an example of how the implementation of point-of-care ultrasound can help differentiate between simple urinary tract infections versus complicated causes of pyelonephritis, more specifically the presence of hydronephrosis that might require urologic intervention if the patient has evidence of sepsis and genitourinary obstruction. In this young patient, with no prior history of urinary tract infections, ultrasound helped us by identifying a condition that if discharged with typical treatment for urinary tract infection could have caused the patient to return with a worsening condition due to the presence of a genitourinary tract obstruction and systemic inflammation [8]. As emergency medicine continues to move toward metrics such as door-to-antibiotic time and door-to-disposition time, the use of a tool that helps the clinician quickly rule out or rule in a diagnosis can be valuable. Point-of-care ultrasound is an integral part of the training required for emergency medicine physicians, and, as more evidence continues to be published regarding the benefits of this technology, it will be just a matter of time before other medical specialties start making point-of-care ultrasound part of their core curriculum. Thus, facilitating early ultrasound training for medical students will give the clinicians of the future the skills and confidence necessary to directly improve patient care in the future.

## Conclusions

Given that, invariably, any cause of genitourinary obstructions leads to hydronephrosis, point-of-care ultrasounds present themselves as a useful tool to quickly evaluate patients with suspected pyelonephritis to efficiently rule out the possibility of complicated infections that may require urgent consultation or intervention. There appears to be more and more emerging evidence that point-of-care ultrasound has the potential to help physicians improve their management of patients diagnosed with sepsis. It seems almost natural that there could be a future protocol for sepsis implementing point-of-care ultrasound. As advances continue in the technology in the field of ultrasound, this instrument should be more readily available for even the most remotely located medical professional. An organized protocol for point-of-care ultrasound in the setting of sepsis could help physicians achieve a timelier diagnosis of some sources of sepsis that might require immediate intervention to improve patient outcomes. There should be a concerted effort by medical school faculty to implement ultrasound early in clinical medical education.
